# The Link Between Obesity and the Skin

**DOI:** 10.3389/fnut.2022.855573

**Published:** 2022-03-10

**Authors:** Razvigor Darlenski, Vesselina Mihaylova, Teodora Handjieva-Darlenska

**Affiliations:** ^1^Department of Dermatology and Venereology, Acibadem Cityclinic Tokuda Hospital, Sofia, Bulgaria; ^2^Department of Dermatology and Venereology, Trakia University, Stara Zagora, Bulgaria; ^3^Department of Pharmacology and Toxicology, Medical Faculty, Medical University, Sofia, Bulgaria

**Keywords:** acne, lichen planus, psoriasis, melanoma, anti-obesity drugs, overweight, bariatric surgery

## Abstract

Obesity as a multi-organ disease that affects the entire human organism. Notably, the skin is no exclusion from this postulate. Skin changes in obese patients have been widely studied with regards to mechanical friction, skin infections, and skin hypertrophic conditions, such as acanthosis nigricans and, most commonly, fibromas (skin tags). Almost 60–70% of obese patients present with a variety of skin changes. Herein, we discuss our own experience and review the complex skin changes in obesity. The role of metabolic syndrome and obesity are responsible for the epidemiological prevalence and are involved in the pathogenesis of chronic inflammatory skin diseases, such as psoriasis, atopic dermatitis, and skin malignancies. Here, we comment on the role of nutritional interventions in these patients as it has been proven that low-calorie diet and weight loss is related to improvement of inflammatory skin diseases. The readership of this paper will receive up-to-date overview on the connection between obesity and the skin that is of a practical importance to any clinician working in the field.

## Introduction

Skin, as the largest organ, accomplishes multiple defensive functions and contributes to the mammalian organism homeostasis. Skin physiology and pathology are influenced by the interplay of hormones, immune signaling molecules, and growth factors. Therefore, skin homeostasis reflects the inner state of the organism (1, 2). For centuries, the skin has been accepted as a “mirror,” in which the health status of internal organs is reflected. Classic examples of skin changes in systemic diseases include paraneoplastic skin syndromes, accumulation disorders (e.g., Fabry disease), and genetic inherited conditions with cutaneous involvement (3).

In the past decades, the concept of obesity, formerly accepted as an isolated condition, has evolved and is now disclosed as a systemic disease with multiple organ and system involvement (4). Numerous co-morbidities beyond the cardiovascular system have been reported, i.e., non-alcoholic fatty liver disease, autoimmune disorders, asthma, atopic dermatitis, and chronic inflammatory conditions such as psoriasis, rheumatoid arthritis, and cancer (5). Despite this, skin changes in obesity have been underestimated, with almost 50% of obese and overweight patients displaying skin comorbidities in relation to a metabolic disorder (6). In addition, a plethora of skin diseases, such as psoriasis, atopic dermatitis (AD), and melanoma, are influenced by comorbid obesity. Considering the increasing pandemic prevalence of obesity and the accessibility of the skin for inspection on the other, skin changes in obesity should be recognized by clinicians.

The aim of this paper is to summarize the current knowledge on the link between obesity and the skin. We describe skin physiology changes in obese and overweigh patients and specific skin manifestations of obesity are disclosed. A review on the affection of chronic skin diseases from comorbid obesity is presented together with the adverse skin reactions to anti-obesity medications.

## Skin Anatomy and Physiology Changes in Obesity and Overweight

The skin of an obese patient is characterized by increased subcutaneous fat, larger skin folds, and higher surface roughness (7–9). Early animal studies in obese-hyperglycemic mice revealed increased skin surface area, collagen abnormalities related to abundance of reducible cross-links, and glycosylated lysine in relation to decreased skin mechanical resilience (10). Reduced expression of certain ceramide types (structural components of epidermal lipids) was observed in obese females (9). This could be of clinical relevance for the development of stretch marks (striae distensae) and impaired wound healing in obesity.

Data on the permeability barrier function, measured by the assessment of the insensible transepidermal water loss (TEWL), is controversial. While increased basal TEWL was disclosed in the adult (11) and children population (12), suggesting increased skin barrier permeability, some patients can present contrasting values (7). This may be due to inconsistencies in the study related to acclimatization of subjects before measurement and the use of closed chamber device. Lower TEWL in patients who are overweight and with low grade obesity was noted in comparison to lean and excessively obese individuals (13). The authors attributed these discrepancies as an adaptation mechanism of the permeability water barrier to weight gain.

Skin dryness is a profound physiologic feature of obese patients’ skin, which has been confirmed by non-invasive measurements of skin electrical conductivity (9, 14). The objective changes correlated well with the self-perception of skin xerosis.

Skin erythema (redness) is increased in obese patients in relation to increased skin blood flow (11). However, several studies contradicted that erythema is, instead, due to decreased skin microcirculation and inadequate vascular reactivity of the skin capillaries that were maximally recruited at baseline (15, 16). This together with the decreased skin lymphatic drainage in obesity could explain the hindered wound healing and the development of stasis conditions and lymphedema.

## Specific Skin Involvement in Obesity

Obesity and overweightness are correlated with specific skin changes. The association between monogenic obesity syndromes, such as Prader–Willy, McCune–Albright, and fragile X syndromes, and skin pathology is beyond the scope of this review. Hence, the readership is referred to the work of Millington for a detailed overview (17). For deductive purposes, we propose the following classification of specific skin changes related to obesity.

### Mechanistic Complications

**FIGURE 1:**
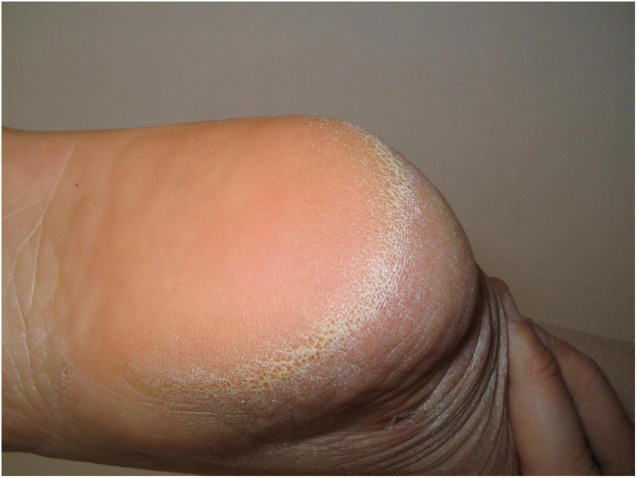
Horseshoe-like plantar hyperkeratosis of the heel.

Analysis of 156 obese patients revealed the plantar “horseshoe-like” hyperkeratosis (Figure 1) as the most typical cutaneous stigma in this population (18). This reaction, together with the decrease of the plantar arch, is a defensive mechanism of the skin against mechanical friction and increased gravity load of the heels. The management of this condition requires topical keratolytic agents (most commonly based on urea or salicylic acid).

**FIGURE 2:**
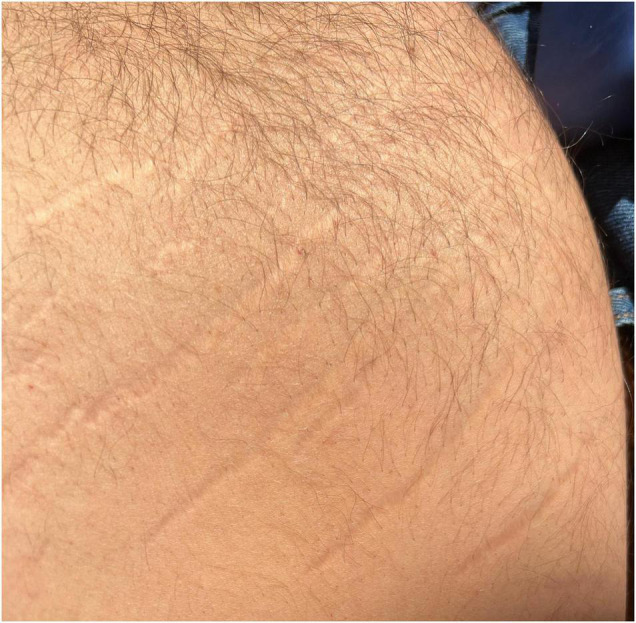
Striae distensae (stretch marks) on the trunk of a patient with obesity.

Striae distensae, also known as stretch marks, results from the mechanical stretching and tearing of dermal elastic fibers (Figure 2). They represent longitudinal atrophic marks most commonly on the breasts, abdomen, tights, and buttocks (8, 18, 19). While the newly appeared lesions are erythematous, with time, they become pearl-colored and atrophic. Such changes could be a manifestation of Cushing’s syndrome and elevated cortisol levels. Hence, patients with striae distensae should seek medical examination. Currently there is no effective treatment for this condition despite the multiple treatment modalities offered by aesthetic dermatologists.

### Skin Changes Due to Hyperinsulinemia and Insulin Resistance

**FIGURE 3:**
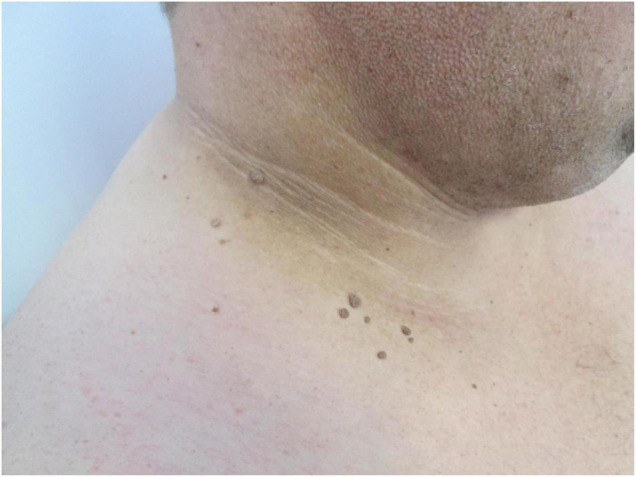
Acanthosis nigricans presented with velvety, brown plaques, and skin tags (fibroma pendulum) on the neck of the patient.

Insulin resistance, a common finding in obese patients (20), has been shown to induce specific skin changes. Acanthosis nigricans (Figure 3), which presents with velvety pigmented and verrucous plaques on the skin folds (neck, axilla, inguinal, and sub-mammary), results from the stimulation of the proliferation of dermal fibroblasts and epidermal keratinocytes by insulin, insulin-like growth factor 1 (IGFR1), fibroblasts, and epidermal growth factors (8, 21). This condition should be distinguished from the obligatory paraneoplastic acanthosis nigricans (22). Weight reduction, topical retinoids, keratolytic agents, and agents targeted against insulin resistance have been shown to be effective in the treatment of this condition (23).

Fibroma pendulum (skin tags, acrochordons) represent pedunculated skin-colored papules, most commonly involving the predilection skin sites for acanthosis nigricans (skin folds) (Figure 3) (24). The number and the distribution of skin tags has been correlated to the degree of hyperinsulinemia (25). The management of fibromas require surgical excision and resides in the field of aesthetic medicine.

Keratosis pilaris, a widely prevalent condition, has been linked to increased body mass index (BMI) (26). The disorder is characterized by tiny keratotic follicular papules on the outer upper arms, buttocks, tights, and the cheeks. Topical moisturizers, keratolytic agents, and retinoids may be helpful in the management of keratosis pilaris (27).

### Skin Conditions Resulting From Hyperandrogenism

Hyperandrogenism, resulting from adipose tissue or ovarian production of androgens, has been linked to virilism, manifested by hirsutism (most often facial), acne, androgenic alopecia, seborrhea, and several syndromes such as the hyperandrogenism, insulin resistance and acanthosis nigricans (HAIR-AN) and seborrhea, acne, hirsutism, alopecia (SAHA) (24, 28). Anti-androgen hormonal therapy is a treatment of choice in these conditions.

### Skin Infections

**FIGURE 4:**
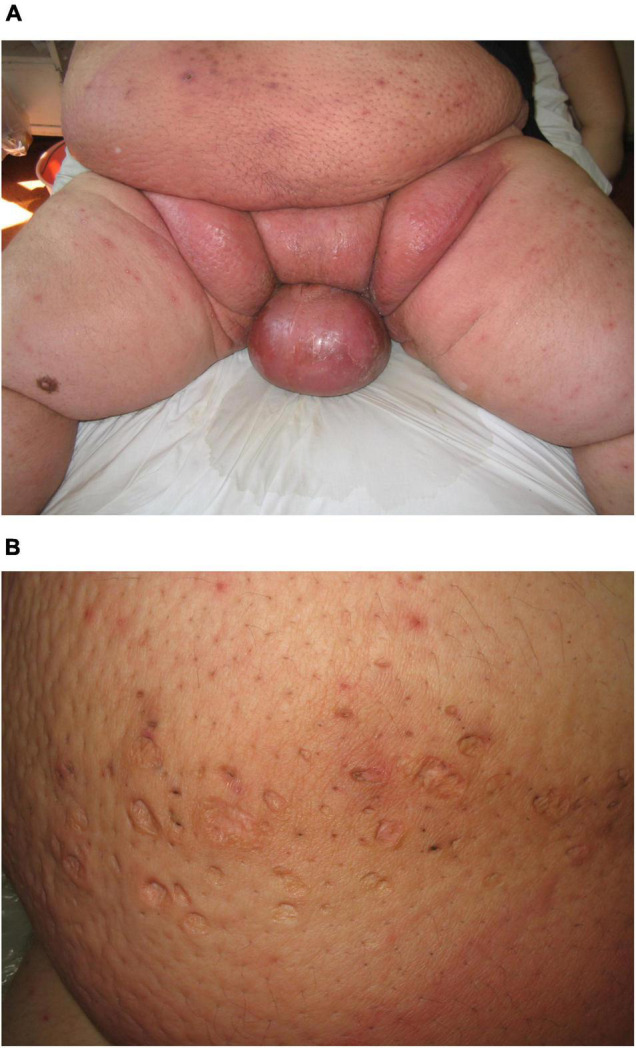
**(A)** Skin bacterial cellulitis of the scrotum and inguinal area of an obese male patient. **(B)** Atrophic round scars, secondary to the resolution of bacterial folliculitis, resembling “moon crater” pattern.

Skin infection complications are associated with obesity and overweightness. Staphylococcal and streptococcal infection of the skin and its appendages is typical (29, 30). In Figure 4A, we present a male obese patient with scrotal bacteria cellulitis (erysipelas) who presented with diffuse erythema and edema. In Figure 4B, the residual changes after repeated bacterial folliculitis can be observed. The pattern resembles a “moon crater” due to the excessive tension forces in the process of resolving Staphylococcal folliculitis.

Intertrigo, a common skin fold (inguinal, axillar, sub-mammary) associated with obesity, is due to the increased friction, occlusion, and maceration in these sites and is most commonly associated with Candida or Gram positive bacteria overgrowth (31). This should not be confused with erythrasma – a corynebacterial skin fold infection also observed in obesity (8).

Onychomycosis, caused by different dermatophyte species, is commonly observed in obese patients. Patients with this condition are noted to present therapy resistance, particularly in obese and overweight patients (32).

### Skin Changes Due to Chronic Venous Insufficiency

**FIGURE 5:**
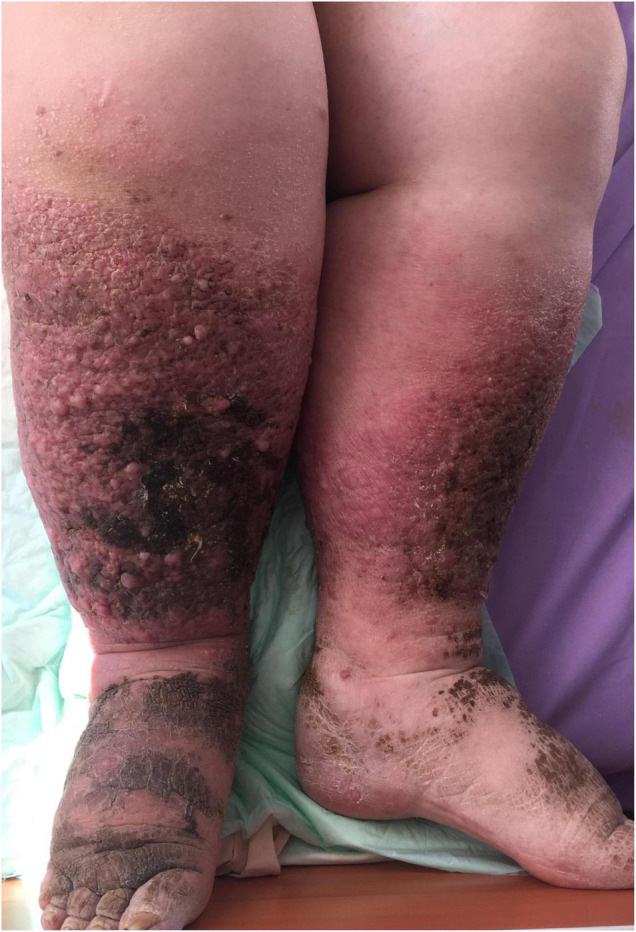
Elephantiasis nostras verrucosa with skin papillomatosis, pigmentation, and soft tissue overgrowth.

The increased intra-abdominal pressure in obese patients results in chronic venous insufficiency (CVI) which manifests as varices, lower limb edema, leg ulcers, and lymphedema (33). In Figure 5, we present a rare case of elephantiasis nostras verrucosa, which resulted from CVI and secondary lymphedema of the lower limbs characterized by epidermal papillomatosis, in a 54-year-old patient with a BMI of 48.5.

## Chronic Skin Conditions Influenced by Comorbid Obesity

In the past decades, the role of obesity in chronic skin conditions such as psoriasis, hidradenitis suppurativa (HS), and atopic dermatitis has been reevaluated. Far from mechanistic association, common pathophysiology mechanisms have been disclosed in chronic inflammatory skin disorders and obesity (34).

### Psoriasis

Psoriasis as a chronic systemic inflammatory disease that shares pro-inflammatory mechanisms with obesity, including Th1 cytokines [interferon-γ, interleukin (IL)-2, IL-12, and tumor necrosis factor (TNF)-α] (35). More recently, the family of adipokines have been involved in the obesity-driven inflammation in psoriasis (36). Obesity is an independent risk factor associated with psoriasis severity and response to systemic therapy (i.e., methotrexate, cyclosporine, apremilast, and biologic agents) (37–39). Finally, dietary interventions and weight loss improve the disease course and severity of comorbid psoriasis (40, 41).

### Hidradenitis Suppurativa

Hidradenitis suppurativa (HS) characterized by chronic inflammatory nodules, cysts, and abscesses, resolves with scar formation and engage the skin folds. Obesity is a risk factor for HS both in children and in adults (42, 43). Weight loss is associated with disease improvement (44), while bariatric surgery has been shown to worsen or induce *de novo* HS (45). A survey revealed that Western diet rich in high glycemic index and fat food exacerbates the course of HS, while fruit and vegetable-rich diets were reported to alleviate HS symptoms. Still, prospective cohort studies are required to evaluate the effect of diet on the disease prognosis.

### Atopic Dermatitis

The link between obesity has been disclosed and reviewed by several studies (46). The severity of atopic dermatitis (AD) correlated well with BMI (47). In addition, children whose mothers exhibited pre-pregnancy obesity and overweightness had a higher risk for AD development in early childhood (48). Plausible explanations for the increased incidence and comorbid state of AD and obesity is the sub-clinical systemic inflammation with BMI increase and the immune modulating properties of adipokines such as leptin, resistin, and ghrelin (49, 50). Weight reduction results in better AD treatment outcomes (47).

### Melanoma

Increased BMI has been revealed as a risk factor for skin melanoma. However, the link is not as strong as in other malignancies (51, 52). In contrast, even a “obesity paradox” was disclosed for melanoma (51). Gut microbiome modulation by diet could be the explanation and, possibly, the link between obesity and melanoma (53). Still, the molecular mechanisms of this interaction remain to be elucidated.

### Miscellaneous

Acne vulgaris is a common disorder with increasing prevalence. It has a certain link to obesity, insulin resistance, and altered peripheral skin receptor sensitivity to sex hormones (54, 55). High glycemic index and Western diet have been linked to acne worsening (56).

Lichen planus, a chronic itchy inflammatory skin disease characterized by flat round and polygonal papules, has been linked to abdominal obesity and other features of the metabolic syndrome (57).

Systemic lupus erythematosus, as an autoimmune disorder, is worsened with the increase in BMI. In addition, physical activity has been shown to ameliorate the pro-inflammatory effect of the higher body mass (58). The link between obesity and autoimmunity has been disclosed in mice. Hence, low-fiber diet could be the milestone in the complex disease interactions (59).

## Adverse Skin Reaction to Anti-Obesity Treatment

The pandemic spread of obesity has led to the development of multiple medications and interventions for its treatment. Orlistat has been reported to induce leukocytoclastic vasculitis (60, 61) and lichenoid skin eruption (62, 63). Dexfenfluramine and phentermine have been linked to the development of rash (2%), alopecia, sweating, urticaria, and pruritus (64). Sibutramine could provoke rash (3.8%), sweating (2.5%), herpes simplex (1.3%), acne (1.0%), and, though rarely, bullous drug eruptions (65). Novel players, such as liraglutide, was shown to induce injection site reactions such as erythema, pruritus, and rash. A single report also disclosed vesiculopustular dermatosis caused by this agent (66, 67). Beyond injection site reactions, semaglutide has been suspected to cause hair loss. However, this was not confirmed by other studies (67, 68). We refer the reader to a former work of our group revealing the adverse skin reactions to metformin, commonly used off-label for the treatment of obesity (69). Rarely, reactions to anti-obesity food supplements have been documented, as in the case of lichen planus pemphigoides (70).

Bariatric surgery induces specific skin manifestations that could be divided into (1) diseases due to metabolic and nutritional disorders, and (2) those derived from cutaneous structural changes after major weight reduction (71). The most specific adverse skin reactions to this intervention include bowel-associated dermatosis and arthritis syndrome, erythematous macular eruption with a neutrophilic infiltrate, non-pruritic papular eruption with IgG and C3 deposition, and erythema nodosum (71, 72). In addition, a new subset of HS associated with malabsorption and deficiency in micronutrients has been described (73). Zinc deficiency resulting from malabsorption in bariatric surgery has been linked to the development of the rare condition known as necrotic migratory erythema (74). Despite the clinical improvement of acanthosis nigricans, the development of alopecia has been described in a cohort of post bariatric surgery in relation to malabsorption (75). In post-bariatric abdominoplasty, delayed wound healing, skin infections, and umbilical necrosis have been shown to be significantly correlated with certain predictors such as initial BMI, type 2 diabetes, tobacco smoking, and the amount and timing of abdominoplasty (76).

## Conclusion

The variety of skin comorbidities in obesity is rich, and their understanding is of relevance both for dermatologists and physicians involved in obesity management. The most common specific stigmata of obesity on the skin include the “horseshoe-like” plantar hyperkeratosis, skin tags (fibroma pendulum), and acanthosis nigricans, with the latter specific for the insulin resistance in obesity. Therefore, these signs are considered relevant to be screened in obesity and overweight patients.

On the other hand, the link between certain chronic skin conditions is a subject of a growing body of evidence. Therefore, patients with chronic inflammatory skin disorders such as psoriasis, AD, hidradenitis suppurativa, and systemic lupus erythematosus should be monitored for obesity with the primary goal of losing weight. Evidence suggests that weight loss could improve the course and severity of chronic skin comorbidities such as AD and psoriasis (47, 77). Missing in the literature is the correlation of the specific types of obese patients (e.g., morbid obesity, abdominal obesity) and specific skin diseases. It is an open question whether there is a threshold on the amount of weight loss to improve skin lesions.

Finally, the safety profile of anti-obese drugs and procedures with regard to the skin and its appendages is enriched by multiple case reports, and real-world post-marketing data will broaden our understanding on the diverse nature of obesity.

## Author Contributions

All authors listed have made a substantial, direct, and intellectual contribution to the work, and approved it for publication.

## Conflict of Interest

The authors declare that the research was conducted in the absence of any commercial or financial relationships that could be construed as a potential conflict of interest.

## Publisher’s Note

All claims expressed in this article are solely those of the authors and do not necessarily represent those of their affiliated organizations, or those of the publisher, the editors and the reviewers. Any product that may be evaluated in this article, or claim that may be made by its manufacturer, is not guaranteed or endorsed by the publisher.
